# The cost of removing pollutants from water using magnetic nanoadsorbents

**DOI:** 10.1007/s11356-026-37527-z

**Published:** 2026-02-26

**Authors:** Mthokozisi Mnguni, Philiswa Nosizo Nomngongo

**Affiliations:** 1 Department of Chemical Sciences, Doornfontein Campus, University of Johannesburg, P.O. Box 17011, Doornfontein, 2028 South Africa; 2 Department of Science and Innovation, National Research Foundation South African Research Chair Initiative (DSI-NRF SARChI): Nanotechnology for Water, University of Johannesburg, Doornfontein, 2028 South Africa

**Keywords:** Economic assessment, Cost estimation, Adsorption cost, Environmental water, Magnetic nanoparticles, Magnetic biochar

## Abstract

Magnetic nanoparticles (MNPs) have enhanced the removal of pollutants from water to circumvent the drastic effects on the environment and living organisms. Despite their extensive application in water remediation studies, little is known about their cost analysis. This study comprehensively and critically discussed the different techniques utilised to evaluate the cost analysis of magnetic nanoadsorbents. Fifty-five research articles were selected after employing a systematic inclusion and exclusion criterion. In the studied research articles, about 76% employed the cost of adsorbent preparation economic evaluation method, 15% utilised the cost of adsorption per volume of sample, and 9% of the studies used the cost of adsorption per gram of adsorbate. The study highlighted methodological heterogeneity in cost analysis and identified gaps and challenges in standardising the cost evaluation techniques, which prevent accurate comparison. Moreover, this study demonstrated that the adsorption cost can be significantly reduced by regenerating the adsorbent; for instance, in one study, the cost was reduced from 0.38 to 0.038 USD due to ten-fold reusability of the adsorbent. A worked example demonstrated ways to accurately determine costs and significantly reduce the costs of treating contaminants from water. This included employing the adsorption cost method, incorporating easily accessible waste materials to MNPs, selecting less energy-intensive synthetic techniques, preparing stable materials to ensure reusability, and using industrial-scale costs. As this is an emerging area of research, this study provides a standardised, step-by-step framework for estimating the costs of pollutant removal from water systems, with relevance to policymakers and real-world industrial applications. Clinical trial number: not applicable.

## Introduction

The pollution of our water systems by organic and inorganic contaminants is a global problem, exacerbating the water scarcity crisis. The high level of water pollution is partly linked to the increased industrialisation. This means industrial wastewater containing organic and inorganic pollutants is not adequately treated by wastewater treatment plants (WWTPs) and ends up in surface water. This phenomenon poses a threat to aquatic life and humans, who rely on surface water as a source of drinking water. Several techniques are employed in WWTPs to remove pollutants, including filtration, coagulation, ozonation, advanced oxidation process, and adsorption (Akintola and Ayankunle 2023). However, much attention has been directed at the adsorption process, owing to its high removal performance and simplicity (Akintola and Ayankunle 2023; Guillossou et al. 2019). The efficiency, rapidness, and costs of a treatment process mainly depend on the adsorbent materials. Magnetic adsorbents emerged as suitable materials due to their high adsorptive performance and ease of separation by an external magnet. This property shortens the treatment process. The cost implications of employing magnetic adsorbents on an industrial scale and in municipal wastewater treatment plants are unclear, as these adsorbents have received limited economic analysis. Hence, the aim of this study was to analyse and review the reported cost analysis (including different methods) of magnetic adsorbents used to remove contaminants from water.

**Fig. 1:**
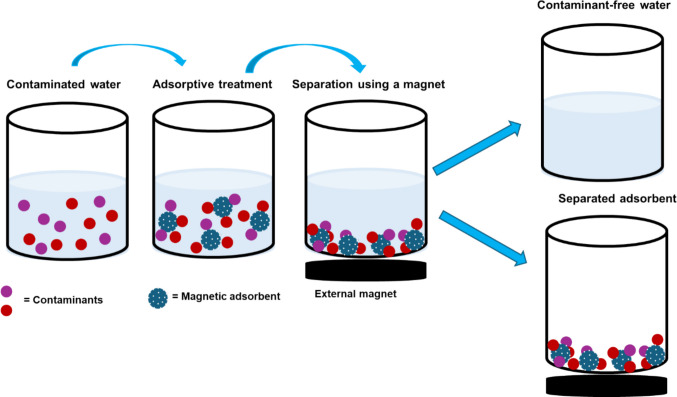
Schematic diagram showing the treatment of contaminated water by magnetic adsorbents

Magnetic iron oxides (Fe_2_O_3_ and Fe_3_O_4_) are the most extensively studied magnetic nanomaterials in water remediation due to their nontoxicity, cost-effectiveness, readily available, and their good adsorption performance (Deligeer et al. 2011). Moreover, the magnetic properties provide easy material separation from the solution after the adsorption process. This aids in saving time and costs as it avoids separation techniques such as centrifugation. As shown in Fig. 1, the magnetic adsorbent was introduced into a sample containing contaminants, followed by agitation by an external force to facilitate adsorption. Once the adsorption was complete, an external magnet separated the magnetic adsorbent with adsorbate from the solution and produced contaminant-free water (Fig. 1). This separation process enabled the desorption of analytes and regeneration of the magnetic adsorbent. This occurrence is instrumental in the reusability of magnetic adsorbents and it saves adsorbent and adsorption costs. Generally, nanomaterials exhibit more surface atoms, a large surface area, high chemical activity, low internal diffusional resistance, high adsorptive capacity, and high surface binding energy (Cui et al. 2006; Khajeh et al. 2013; Khajeh and Sanchooli 2011; Liu et al. 2005). Consequently, they have an enhanced accessible surface, affinity, number of active sorption sites, and surface energy for the targeted analytes, making them attractive in adsorption studies.

Magnetic nanoferrite, MFe_2_O_4_ (M=Mn, Mg, Ni, Co, etc.), is another group of magnetic nanomaterials extensively used in water remediation studies as adsorbents (Amar et al. 2024; Mishra et al. 2020; Tahar et al. 2019). This owes to their ease of preparation, low toxicity, high physical and chemical stability, large adsorption capacity, and moderate saturation magnetisation (which makes it easy to separate from complex wastewater by applying an external magnetic field). Unlike Fe_3_O_4_, MFe_2_O_4_ remains stable even in acidic solutions (pH 2.0–6.0), rendering it applicable for a broad pH spectrum (Reddy and Yun 2016). These properties make magnetic nanoferrites more attractive for hybridisation with other materials to form nanocomposites with enhanced adsorptive performance.

Magnetic nanoparticles (MNPs) are usually functionalised and combined with other materials that exhibit good adsorptive properties to enhance removal performance by introducing more adsorptive sites and ensuring selectivity. Various materials have been used to form magnetic nanocomposites, such as SiO_2_ (Youssif et al. 2024), metallic oxides (Deng et al. 2019), graphene (Alishiri et al. 2023; Kaur et al. 2022), metal-organic framework (Li et al. 2025), biopolymers (Pooladi & Bazargan-Lari 2023), biowaste (Chander et al. 2024), and biochars (Bai et al. 2022; Le et al. 2023), which brings multiple functional groups. Plant-based raw materials, biowaste, and biochars are obtained at a cheap cost or for free; they also significantly reduce the overall treatment costs of contaminants. These materials also make the nanocomposites more environmentally friendly and greener as they use natural and biodegradable materials. The choice of synthetic methods for magnetic materials also significantly influences the cost of adsorbents and the adsorption process. For instance, microwave-assisted, sonochemical, thermal, and pyrolysis methods require energy-intensive instruments such as microwaves, ultrasonicators, ovens, and furnaces.

For years, researchers focused on only developing best-performing adsorptive materials until recently, when emphasis was also placed on synthesising cheap materials that can be applied on a large industrial scale. Concurrently, there is also a focus on reducing environmental waste by converting waste materials and plant-based raw materials into environmentally friendly value-added adsorbent materials. However, as this sphere of research is still emerging, there is heterogeneity in the methods employed to estimate adsorbent preparation costs across various studies. The major inconsistencies were in the selection of parameters that contribute to the economy of the material, such as the cost of chemicals, energy consumption, and water bills. Some studies excluded parameters that significantly influence the cost of preparing material in their economic analysis; these factors will be highlighted in more detail in the subsequent sections. This made it difficult to fairly compare the costs of adsorbents and treatment costs to select the best-performing magnetic material at a lower price. The aim of this study was to review existing cost analysis techniques for magnetic adsorbents and discuss the heterogeneity in the current techniques.

## Methodology

**Fig. 2:**
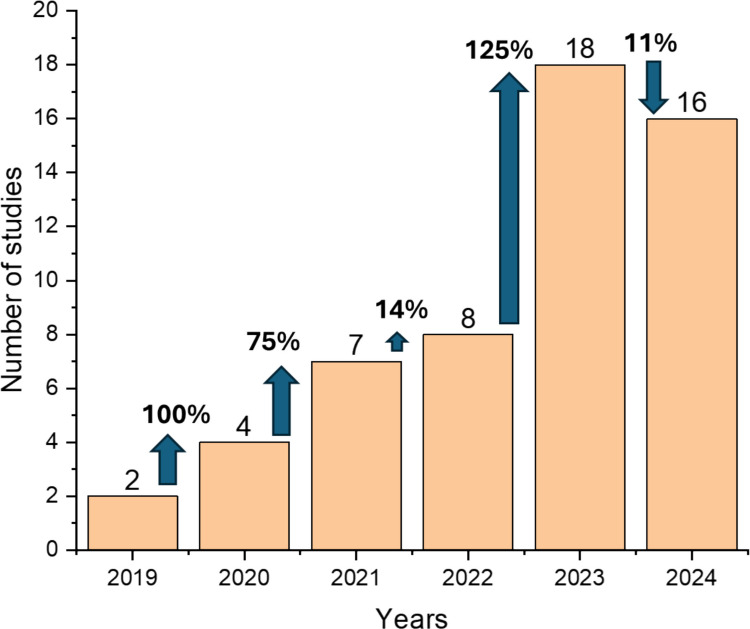
Number of published studies (and the increase rate in each year) that included cost analysis of magnetic adsorbent in the adsorption of pollutants from water from 2019 to 2024

This study entailed a comprehensive and critical review of research articles that synthesised magnetic adsorbents for water and conducted cost analysis. The magnetic adsorbents included MNPs and their composites (magnetic nanocomposites). A comprehensive literature search was conducted on Google Scholar and Web of Science, and it focused on published research articles in the English language between 2019 and 2024. This study used a combination of search terms, including “cost analysis of magnetic adsorbents”, “economic assessment of magnetic adsorbents”, and “cost assessment of magnetic adsorbents”. The generated articles from the two search engines were screened using titles and abstracts to remove duplicated articles. Hundreds of articles were generated, which also included studies that prepared magnetic adsorbents (without cost analysis), employed cost analysis of general adsorbents (not magnetic adsorbents), and cost estimation/economic assessment/cost assessment of magnetic adsorbents. Studies that included magnetic adsorbents and cost assessments as per our search terms were screened using title, abstract, and synthesis methodology. The remaining studies that did not meet the aforementioned criterion were excluded. Figure 2 illustrates the annual publication of articles relevant to this; a total of 55 research articles were used in this review study. These types of studies have been increasing gradually until 2023, which showed a steep increase (125% increase rate). In 2024, the number of studies slightly decreased by 11%.

## Synthesis of magnetic adsorbents

### Preparation of magnetic nanoparticles

Several chemical-based preparation methods for generating magnetic nanoparticles include co-precipitation (Mohammadi et al. 2021), sol-gel (Amar et al. 2024), microwave-assisted (Lahlahi-Attalhaoui et al. 2024), and thermal decomposition (Klekotka et al. 2024). Co-precipitation technique, which was first reported by Massart, is the simplest and most commonly employed synthetic method to prepare magnetic nanoparticles; this is owed to its easy upscalability and ability to control the particle size and physicochemical properties (Bee et al. 1995; Massart 1981; Niculescu et al. 2022; Wei et al. 2012). For instance, when preparing Fe_3_O_4_ via the co-precipitation method, ferric salt and ferrous salt are dissolved and mixed in a 2:1 ratio under an inert atmosphere. This step is then followed by the addition of an alkaline solution to facilitate the precipitation of the black magnetite. After precipitation, MNP can be separated from the mother solution by applying an external magnet and decanting the mother liquid. This approach is easy and economical as it avoids energy-intensive and expensive centrifugation processes. The physicochemical properties of MNPs can easily be adjusted by controlling the stirring rate, temperature, and pH of the solution (Sirivat and Paradee 2019; Vallejo-Espinosa et al. 2024). Although this method is simple and time efficient and produces a high yield, it suffers from generating a wide range of nanoparticle sizes and also requires a strong and hazardous alkaline solution (Niculescu et al. 2022). As a result, alternative synthetic procedures are continuously developed and improved to counter the drawbacks associated with the co-precipitation method.

Thermal decomposition is another traditional synthetic method of producing MNPs. This technique entails treating organometallic iron precursors, such as iron pentacarbonyl, iron oleate, and iron acetylacetonate, with high temperatures in the presence of organic solvents exhibiting high boiling points and surfactants (Klekotka et al. 2024; Niculescu et al. 2022). Thermal decomposition is famous for producing MNPs with uniform particle sizes, high crystallinity, and isolation (Liu et al. 2020). However, high boiling-point organic solvents are expensive and hazardous, thus making the process environmentally unfriendly and negatively impacting the cost of applying MNPs in water remediation. Moreover, thermal decomposition requires high energy-consuming instruments such as furnaces, which enhances the production cost of MNPs.

The microwave-assisted synthetic approach is emerging as a fast and environmentally friendly alternative to preparing MNPs. Here, microwave irradiation is used as a source of thermal energy instead of conventional heating, which ensures a high yield of MNPs with particle sizes ranging from 5 to 7 nm (Niculescu et al. 2022). Several MNPs have been prepared via microwave-assisted methods, including CuFe_2_O_4_ (Nguyen et al. 2025), ZnFe_2_O_4_ (Darcheville et al. 2024), NiZnFe_2_O_4_ (Fedorchuk et al. 2024; Lahlahi-Attalhaoui et al. 2024), and CoFe_2_O_4_ (Ayyıldız et al. 2023); they enjoy applications in various fields. Despite the excellent advantages associated with the method, it requires highly specialised and expensive equipment. In this case, there is a contradiction between the greenness of the method and its high cost.

### Synthesis of magnetic nanocomposite

**Scheme 1:**
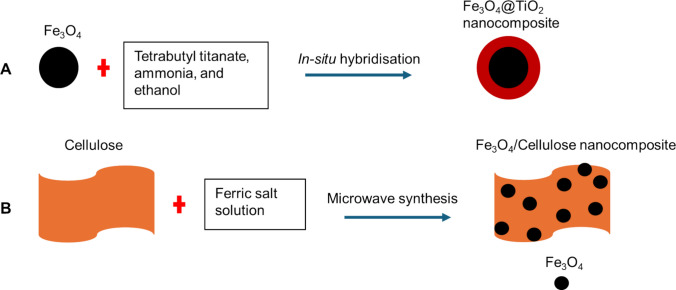
Schematic representation showing two different types (A and B) of hybridisation of MNPs

MNPs are hybridised through functionalisation and combination with other materials to generate magnetic nanocomposites with enhanced properties. There are several routes in preparing magnetic nanocomposites. One route entails preparing MNPs first and then synthesising the second material in the solution containing MNPs, which usually yields core@shell nanocomposite. For instance, Deng et al. engineered a Fe_3_O_4_@TiO_2_ sheet-like nanocomposite in which Fe_3_O_4_ nanoparticles were prepared via the hydrothermal method, and then coated with TiO_2_*in situ* via a sol-gel technique (Deng et al. 2019). The magnetic components formed a core and TiO_2_ was a shell in the core@shell nanocomposite, as shown in the schematic diagram (Scheme 1A). In another study, Youssif et al. synthesised amino-functionalised Fe_2_O_3_@SiO_2_ (Fe_2_O_3_@SiO_2_-NH_2_) via a multistep procedure (Youssif et al. 2024). This involved fabricating Fe_2_O_3_ first, followed by dispersing it in an ethanol-water solution and coating it with SiO_2_ through a sol-gel Stöber method, and subsequently functioning with 3-aminopropyltriethoxysilane (Youssif et al. 2024). The nanocomposite was core@shell nanospheres, as demonstrated in the schematic diagram (Scheme 1A).

Another route involves synthesising MNPs and the material of interest to form a nanocomposite separately and dispersing them in one solution to facilitate bond formation (Alishiri et al. 2023; Kaur et al. 2022). Here, MNPs are usually anchored on the surface of the material, as demonstrated in the schematic diagram (Scheme 1B). Sometimes, the precursors for the magnetic component and the material that forms part of the nanocomposites are mixed, and both components are generated simultaneously. For example, Mkumbuzi et al. employed a microwave-assisted approach to generate Fe_3_O_4_/carbonised cellulose nanocrystals nanocomposite (Fe_3_O_4_/cCNCs) (Mkumbuzi and van Zyl 2024). Here, ferric salt and sugarcane bagasse were mixed as precursors and introduced in the microwave to produce Fe_3_O_4_/cCNCs nanocomposite within 3 min (Mkumbuzi and van Zyl 2024). In these nanocomposites, the MNPs are embedded on the surface of the cCNCs through chemical bonds, as shown in Scheme 1B. Moreover, this study employed biowaste, making the nanocomposite sustainable and greener since it avoided the discarding of sugarcane bagasse while producing value-added materials.

## Cost analysis of magnetic adsorbents

There is an upsurge of interest in analysing the cost of adsorbents designed to remove contaminants from our water systems. This will aid policymakers in making informed decisions when weighing the choice of the most effective material against the economic factors involved. It should be noted that relying solely on the manufacturing cost of adsorbents to estimate the feasibility of employing the removal method in municipalities and industries is inaccurate. Several factors that contribute to the overall cost need to be considered, including the availability and accessibility of raw materials to prepare the adsorbent, the removal performance, and the reusability of the adsorbent. This section discusses some of the most used cost estimation approaches for magnetic adsorbents and reflects on the advantages and shortcomings associated with some of them.

### Costs per preparation of adsorbents

Several research studies estimated the economic feasibility of producing adsorbents for water remediation based on the costs of fabricating the adsorbent material. However, researchers and regulators have no consensus on the standard parameters to be included when estimating the cost of synthesising the adsorbents. Based on the studies presented in Table 1, the cost estimations included the cost of synthetic chemicals, raw materials (transportation, purification, size reduction, and carbonisation), electricity, and water. The energy cost emanates from instruments such as hot air ovens, orbital shakers, muffle furnaces, tubular furnaces, ultrasonic mixers, and magnetic stirrers. Despite most of these factors being involved in preparation processes across the presented studies, their inclusion in the economic estimation was not standard. This part of the study analysed and discussed the economic estimations of magnetic adsorbents presented in Table 1.

**Table 1:** The preparation cost of magnetic adsorbents and their adsorptive removal performance for targeted analytes from water

Material	Magnetic component	Analytes	% removal	Adsorption capacity/mg g^−1^	Costs of sorbent/USD kg^−1^	Ref^a^
MagA^b^	Fe_3_O_4_	Pb(II)	> 90	158.7	6.70	(Akar et al. 2022)
MgF-NPs^c^	MgFe_2_O_4_	Malachite Green	> 90	487.6	50.88	(Das et al. 2021)
CT@Fe_3_O_4_	Fe_3_O_4_	U(VI)	99	45.50	14.03	(Das et al. 2023a )
MZ@β-CD-GA	Fe_2_O_3_	Levofloxacin	89–98	145.0	2131.5	(Mpelane et al. 2023)
MCPEI	Fe_3_O_4_	Anionic reactive black 5	100	330.0	532	(Nordin et al. 2021)
MNCDES	Fe_3_O_4_	Malachite Green	92.7	87.72	0.00180	(Sadiq et al. 2021)
MnFe_3_O_4_/CS	Fe_3_O_4_	Cu(II)	-	62.30	3.30	(Zhang et al. 2021)
		Cd(II)		60.60		
MF-CMS	Fe_2_O_3_	Tetracycline antibiotics	85.9–96.5	64.93–94.63	5.91	(Zhang et al. 2023a)
MIP-MBC	Fe_3_O_4_	Sulfamethoxazole	95.7	25.65	0.100	(Li et al. 2023)
MJRB	Fe_2_O_3_/Fe_3_O_4_	Ni(II)	98.1	18.11	1.66	(Isaac et al. 2023)
		Cu(II)	98.3	16.83		
Zr@MPN-PEI	Fe_3_O_4_	PO_4_^2−^	> 70	36.60	0.501	(Aryee et al. 2023)
MAG–OSBC	Fe_3_O_4_	Clofazimine	98.6	174.0	126.45	(El‐Azazy et al. 2021)
SACFe^d^	Fe_2_O_3_	Sulfamethoxazole	95.4	58.44	2850.3	(Prasannamedha et al. 2022)
BTSFe^e^	Fe_2_O_3_	Ofloxacin	96.0	19.74	2.70	(Singh and Srivastava 2020)
Fe_3_O_4_@DAL	Fe_3_O_4_	Pefloxacin, lomefloxacin, enrofloxacin, and difloxacin	> 80.0	19.2–33.7	275.58	(Hou et al. 2022)
NH_2_-CF-CB	CoFe_2_O_4_	Malachite Green	> 80.0	357.2	47.60	(Sahare et al. 2023)
		Cu(II)		158.7		
CoFe_2_O_4_	CoFe_2_O_4_	Thiazole Yellow G	99.0	67.06	267.42	(Patil and Behera 2023)
		Alizarin Yellow R	93.0	60.19		
NiFe_2_O_4_	NiFe_2_O_4_	Thiazole Yellow G	96.9	63.04	69.99	
		Alizarin Yellow R	92.0	55.71		
MgFe_2_O_4_-PANI-NC	MgFe_2_O_4_	Methyl Red	90.2	98.04	100.60	(Das and Debnath 2023)
FAM-MCA/Fe_3_O_4_	Fe_3_O_4_	Victoria Blue	> 90.0	279	6.64	(Sheerazi et al. 2024)
MGO@TNs	Fe_3_O_4_	Pb(II)	99.8	322.7	29.76	(Yang et al. 2020)
ZnO-Fe_3_O_4_	Fe_3_O_4_	Methyl blue	-	330.0	1.50	(Cheng et al. 2019)
MGO-NH-SH^f^	Fe_3_O_4_	Hg(II)	> 90.0	208.8	38,554	(Kazemi et al. 2019)
Fe_3_O_4_@SiO-NH-SH^g^	Fe_3_O_4_		> 90.0	176.8	20,202	
MMPC/Cyc-Chit^h^	Fe_3_O_4_	Three fluoroquinolones	> 90.0	130–195	1324.9	(Mashile et al. 2020)
MNSB^i^	Fe_2_O_3_	Tetracycline hydrochloride	96.3	143.9	52.59	(Zhang et al. 2024a)
GSMB2^j^	Fe_2_O_3_	Cr(VI)	-	7.61	2.44	(Zhang et al. 2024b)
Fe_3_O_4_-RAC^k^	Fe_3_O_4_	Methylene blue	95.2	32.25	22.29	(Vaddi et al. 2024)
MBC-IO^l^	Fe_3_O_4_	Pb(II)	93.5	504	1.912	(Latifian et al. 2024)
POM-MBC^m^	Fe_3_O_4_	Metronidazole	94.3	78.45	80.03	(Mohammadian et al. 2024)
CHB-Fe-CS^n^	Fe_2_O_3_	Zn(II)	> 85.0	270	10 430	(Sopanrao and Sreedhar 2024)
γ-Fe_2_O_3_-LSB^o^	Fe_2_O_3_	Methylene blue	98.0	54.55	1.88	(Mishra et al. 2024)
PVA-MH^p^	Fe_2_O_3_/Fe_3_O_4_	Cd(II)	95.7	13.8	8.38	(Karlıdağ et al. 2024)
GSH/Fe_3_O_4_@GO^q^	Fe_3_O_4_	Ag(I)	-	755	1900	(Zounia et al. 2024)
FCZ@C-600^r^	Fe_3_O_4_	Dimethylhydrazine	-	185.7	660.24	(Su et al. 2024)
Mag@GAS^s^	Fe_3_O_4_	Tylosin tartrate	80.6	188.6	4.30	(Li et al. 2024)
Fe_3_O_4_	Fe_3_O_4_	Cr(VI)	100	91.15	110	(Yadav et al. 2024)
Fe_3_O_4_/NiO	Fe_3_O_4_		100	150	149.07	
Pan-MgF-NC^t^	MgFe_2_O_4_	Cationic brilliant green dye	90.3	294.1	85.36	(Das et al. b)
Fe-MC3-1000^u^	Fe_2_O_3_/Fe_3_O_4_	Congo red	97.2	972.1	20.83	(Zhang et al. b)
AF-Fe_3_O_4_^v^	Fe_3_O_4_	Cr(VI)	-	212.1	16.19	(Luo et al. 2023)
DETA/SiO_2_/Fe_3_O_4_^w^	Fe_3_O_4_	Cu(II)	99	13.46	1.3	(Chen and Chang 2022)
P3ABA/GO/CoFe_2_O_4_^x^	CoFe_2_O_4_	Congo red	98	153.9	2906	(Babakir et al. 2022)
Wheat bran/Fe_3_O_4_	Fe_3_O_4_	Methylene blue and methyl violet	97.5 and 98.8	46.08 and 51.28	8.00	(Pooladi et al. 2021)
0.3Ma-MgMnLDO-a^y^	Fe_3_O_4_	Cd(II)	99.0	422.6	39.0	(Chen et al. 2020)

^a^References

^b^Magnetically functionalised alunite

^c^Magnesium ferrite nanoparticles

^d^Magnetic sodium alginate beads

^e^Iron oxide nanoparticle incorporated on mesoporous biochar derived from textile mill sludge

^f^Thiol-functionalised graphene oxide

^g^Thiol-functionalised magnetic graphene oxide

^h^Magnetic mesoporous carbon/β-cyclodextrin–chitosan

^i^Magnetic nitrogen-doped biochar

^j^Green synthesis magnetic biochar

^k^Fe_3_O_4_ nanoparticles with rice husk activated carbon

^l^Magnetic biochar-iron oxide

^m^Polyoxometalate-modified magnetic biochar

^n^Chitosan-magnetic biochar composite

^o^Magnetic *Lagenaria siceraria* biochar

^p^Poly(vinyl alcohol)-based magnetic hydrogel

^q^Magnetic graphene oxide functionalised with glutathione

^r^ZIF-8-derived magnetic porous carbons pyrolysed at 600 ℃

^s^Magnetic hydrogel

^t^Polyaniline-based magnesium ferrite nanocomposite

^u^Magnetic sucrose-derived Fe-containing mesoporous carbon composites

^v^Amino-functionalised Fe_3_O_4_

^w^Magnetic adsorbents with amino-functionalised polymer

^x^Poly(3-aminobenzoic acid/graphene oxide/cobalt ferrite) nanocomposite

^y^Multifunctional magnetic MgMn-oxide composite

#### Energy cost

Some studies estimated sorbent cost using only the cost of chemicals employed in the preparation procedure. For instance, Yang et al. calculated the cost of preparing magnetic graphene oxide-titanate composites (MGO@TNs) using only the cost of chemicals involved in the synthetic method (Yang et al. 2020). Another study by Sheerazi et al. also employed the same approach when estimating the cost of preparing magnetic folic acid-mannitol/mosquito coil ash nanocomposite (FAM-MCA/Fe_3_O_4_) to remove Victoria Blue from water (Sheerazi et al. 2024). Both studies underestimated the cost by not including the energy cost associated with heating and drying the material using the oven, compared to other studies in Table 1 that considered the cost of electricity. Electricity can be a significant factor in estimating costs, as it is expensive in many regions of the world. Most synthetic procedures employ an oven to remove moisture, which consumes high energy. This is demonstrated in a study by Das et al., where the cost of oven-drying magnesium ferrite nanoparticles (MgF-NPs) was 52% of the total estimated preparation cost (50.88 USD kg^−1^) (Das et al. 2021). Moreover, 31% of the total cost was attributed to the calcination step by the muffle furnace, bringing the total energy cost to 83%. Elsewhere, the overall cost of preparing magnesium ferrite and polyaniline nanocomposite (MgFe_2_O_4_-PANI-NC) was 100.60 USD kg^−1^ and 26.7% of it was ascribed to the energy utilised for drying and calcination (Das and Debnath 2023). In a different study, 23.5% of the total cost (5.91 USD kg^−1^) was due to the energy used for oven drying, excluding the energy attributed to the carbonisation of raw materials (Zhang et al. 2023a). It is evident that the exclusion of energy cost significantly underestimates the total cost of preparing adsorbing material. This underestimation can eventually lead to the failure of water remediation projects, especially on an industrial scale and in municipal wastewater treatment plants.

The high energy costs have prompted researchers to search for cheaper and cost-free alternative methods to reduce the cost of preparing adsorbents. Other studies applied air (Prasannamedha et al. 2022) and sunlight (Isaac et al. 2023; Singh and Srivastava 2020) as drying methods to remove moisture from raw materials instead of using a high energy-consuming hot air oven. Isaac et al. (2023) dried the washed *Juglans Regia* nut shells in the sun for 3 days as a cost-cutting measure. Three days is a long time for a single step and in a multiple-step synthetic process, which might affect adsorbent availability in industrial-scale applications. Secondly, this drying method depends on sunny weather conditions and it is hampered in winter seasons. This may hugely affect the production of the magnetic adsorbent. Cost-cutting measures should never be taken at the expense of time, economy, or the quality of the material. For instance, incomplete drying of the material under air can cause oxidation during the carbonisation step and produce a carbon material containing ash, thus affecting the removal of pollutants from water. Additionally, the presence of moisture can lead to an inaccurate weight measurement of the adsorbing material. Subsequently, it affects the adsorption capacity of the material since it is dependent on the adsorbent mass. The study by Isaac et al. further reduced the cost by applying manual handshaking instead of a magnetic stirrer (which consumed high energy) (Isaac et al. 2023). Another energy-saving method entails applying a manual size reduction technique to raw materials (Isaac et al. 2023; Singh and Srivastava 2020), as opposed to using the energy-consuming mechanical and analytical mill as seen elsewhere (Aryee et al. 2023; Akar et al. 2022). Therefore, cutting the cost of preparing the material is vital, but the substituting techniques should never alter the performance of the adsorbent.

#### Water cost

Water, an essential factor in most chemical and physical processes, is often ignored when estimating the overall cost of adsorbents. This leads to a serious underestimation of the remediation process, especially in regions where water is scarce and expensive. Das et al. included the cost of water when estimating the cost of preparing *Cinnamomum tamala* leaf extract-coated magnetite nanoparticles (Fe_3_O_4_@CT) (Das et al. 2023a). In their study, 6 USD kg^−1^ of the total cost of 14 USD kg^−1^ was attributed to water (Das et al. 2023a). It is evident that water is a critical factor in cost analysis, and a process relying on deionised or ultrapure water might incur additional treatment costs beyond the standard tap water bill. Isaac et al. (2023) washed the shell waste with deionised water to remove dirt and dust but allocated zero water cost in the cost analysis. This is an apparent underestimation, as it does not include the cost of tap water supplied by the government and purified using a system that operates under electricity. Several other studies presented in Table 1, including El‐Azazy et al. (2021), Singh and Srivastava (2020), and Sahare et al. (2023), excluded the cost of water utilised in the preparation step. Aryee et al. (2023) included the expense of tap water used to clean the raw material and ultrapure water for rinsing the adsorbent; the latter contributed 100 times more to the adsorbent cost than the former. Moreover, ultrapure water contributed 20% of the overall cost. This phenomenon bolsters the argument that neglecting the cost of water, such as purified water, underestimates the costs of adsorbents.

#### Labour cost

Labour is one instrumental aspect in cost analysis that is usually overlooked. From the material synthesis to their application in removing contaminants, all these steps require human resources, which must be financially compensated. The complexity of this parameter may be a justification for excluding it. It is difficult to determine the salary package for an employee who performs the synthetic procedure for the types of materials presented in Table 1. Some studies use the minimum wage in their countries, calculated on a daily basis, to estimate labour. Das et al. (2023a) utilised this technique and found that labour would cost about 5.20 USD kg^−1^, 37% of the overall cost. The good thing is that this is the only parameter most likely to remain constant even when the material production is increased to an industrial scale.

#### Waste materials as cost-cutting measure

The chemicals/reagents involved in preparing the magnetic material are usually responsible for the high cost of the adsorbents. For example, El‐Azazy et al. (2021) prepared magnetic olive stones biochar (MAG-OSBC) as a magnetic adsorbent, which costs 126.45 USD kg^−1^. However, 88% of the total cost was attributed to the cost of reagents used to prepare the magnetite and thus the magnetic composite. Therefore, it is vital to find cheaper sources of magnetic materials for magnetic adsorbents to compete with existing materials in the market and be suitable replacements in industrial applications. Mpelane et al. (2023) proposed an exciting alternative to significantly reduce the cost of magnetic adsorbents since their proposed magnetic zeolite@β-cyclodextrin-gum Arabic nanocomposite (MZ@β-CD-GA) costs 2131.5 USD kg^−1^. This was considerably higher compared to other studies presented in Table 1. They suggested using fly ash (waste material) as a source of zeolites and acid mine drainage as a source of magnetite to avoid purchasing expensive chemicals for preparing zeolite and magnetite. This is an excellent idea, especially in developing countries like South Africa, which constitute major global mining operations and have abundant acid mine drainage. In addition to reducing the preparation costs of magnetic adsorbents, it also aids in solving the negative environmental impacts of acid mine drainage (Baloyi et al. 2023). There is an upsurge of interest in converting waste into value-added products to cut costs and improve the adsorptive capabilities of existing materials.

The high cost of magnetic nanoparticles has triggered a widespread investigation into suitable, effective, and cheap carbon-based materials to form magnetic nanocomposites. Consequently, most studies presented in Table 1 used plant-based raw materials (Aryee et al. 2023; Das et al. 2023a, b; Hou et al. 2022; Mpelane et al. 2023; Nordin et al. 2021; Sadiq et al. 2021; Zhang et al. 2021), and biochars (El‐Azazy et al. 2021; Isaac et al. 2023; Li et al. 2023; Mashile et al. 2020; Prasannamedha et al. 2022; Singh and Srivastava 2020; Zhang et al. 2023a), to form composites with magnetic nanoparticles. This was aimed at enhancing the adsorption performance and reducing the overall cost of the adsorbents. The latter is due to the fact that most biowaste is free of charge and is abundantly available. The advantage of this combination is that the nanocomposites still possess excellent, easily separable properties. At the same time, the cost is reduced since a fraction of magnetite is used in the application compared to employing 100%. The preparation of magnetic nanosized chitosan deep eutectic solvents (MNCDES) (Sadiq et al. 2021), chitosan-coated manganese ferrite nanocomposite (MnFe_2_O_4_/CS) (Zhang et al. 2021), magnetic functionalised carbon microsphere (MF-CMS) (Zhang et al. 2023a), molecular imprinting functionalisation of magnetic biochar (MIP-MBC) (Li et al. 2023), magnetic biochar derived from *Juglans regia* (MJRB) (Isaac et al. 2023), and zirconium functionalised magnetic peanut husk crosslinked with polyethyleneimine (Zr@MPN-PEI) (Aryee et al. 2023), produced cheap nanocomposites with a relatively low price range of 0.00180–5.91 USD kg^−1^, as seen in Table 1.

Studies by Sahare et al. and Patil et al. are good examples to demonstrate how the combination of magnetic materials with relatively cheap biomaterials (or biowaste) reduces the cost of the adsorbent. Patil and Behera (2023) synthesised cobalt nanoferrites (CoFe_2_O_4_) as an adsorbent to remove Thiazole Yellow G and Alizarin Yellow R at a cost of 267 USD kg^−1^. On the other hand, Sahare et al. (2023) incorporated CoFe_2_O_4_ with amino silane and chitosan derived from shrimp shells to form a nanocomposite (NH_2_-CF-CB) to remove Malachite Green and Cu(II) from water. The adsorbent cost was approximately 5.5 times lower than that of CoFe_2_O_4_ as an adsorbent, yet it still achieved high removal performance and large adsorption capacities. The comparison of these studies in terms of costs aided in justifying the upsurge in interest in integrating magnetic materials with readily available and cheap materials to cut costs and enhance adsorption performance.

Some magnetic adsorbents were expensive despite using biomaterial, which is mainly attributed to the additional expensive chemicals they employed. Exemplary, cellulose-containing magnetic-cellulose-polyethyleneimine (MCPEI) was 532 USD kg^−1^, but 62% of the total cost was ascribed to polyethyleneimine compared to 0.43% and 3.2% attributed to cellulose and magnetite, respectively (Nordin et al. 2021). Polyethyleneimine was added to introduce amine groups to facilitate electrostatic attraction with anionic pollutants. This phenomenon proves hybridisation reduces costs and introduces new functional groups that facilitate selective interactions, target specific contaminants, and improve water remediation. Another excellent example of this was the coating of Fe_3_O_4_ with dealkaline lignin (DAL), which introduced functional groups such as C-O, C=O, O-H, and C=C, which were responsible for the π-π electron-donor-acceptor (EDA) interaction between aromatic and carbonyl groups of Fe_3_O_4_@DAL and fluoroquinolone antibiotics (Hou et al. 2022). This interaction contributed to the high removal percentage, as seen in Table 1.

The raw materials from biowaste are usually free of charge or cost close to nothing. Biochar has been reported to exhibit low cost, energy usage, and greenhouse gas emissions during its preparation compared to commercial adsorbent (activated carbon) (Akintola and Ayankunle 2023). Therefore, materials derived from biowaste are suitable alternatives to reduce adsorption costs. The only costs associated with waste-based raw materials are collection and transportation (Aryee et al. 2023; Hou et al. 2022; Li et al. 2023; Zhang et al. 2021), cleaning and sometimes size reduction, and carbonation (Isaac et al. 2023; Li et al. 2023). However, some studies overlook the cost of transporting and preliminary processing waste materials in their adsorbent cost estimation. Zhang et al. (2021) prepared MnFe_3_O_4_/CS from waste shrimp shells to remove copper and cadmium from water and sediments, which showed good adsorption capacities, as shown in Table 1. Notably, the cost of collecting, transporting, dismantling, purifying, drying, and size controlling the waste shrimp shells was 1.4 USD/kg which was about 42% of the total cost of the magnetic adsorbent (Zhang et al. 2021). This information indicates that Singh et al. might have significantly underestimated the cost of preparing BTSFe since they excluded the cost of transporting raw sludge from the textile industry and washing it with distilled water (Singh and Srivastava 2020). Again, this emphasises the discrepancies in how different studies estimated the cost of magnetic adsorbents in Table 1, thus making it difficult to compare them and choose the best-performing material at the lowest cost. Life-cycle assessment of waste materials and their impact in the environment and cost magnetic nanoadsorbents should be standardised.

#### Overhead cost

Some studies included overhead costs of 10% of the net economy of preparing the magnetic adsorbents, which also covers any losses (Isaac et al. 2023; Li et al. 2023; Nordin et al. 2021; Zhang et al. 2023b, 2021). This means that the costs presented in Table 1 are not fairly comparable as other studies excluded this parameter from their economic analysis. These discrepancies intensify the need to formulate a standard procedure for estimating adsorbent costs for policymakers and industries to make informed decisions when selecting the best adsorbents in wastewater treatment. Additionally, this method of estimating the economic aspect of magnetic adsorbents by only focusing on the synthesis cost is insufficient. It does not include the performance of materials in removing contaminants from water. This aspect is essential in comparing the proposed materials fairly against commercially available adsorbents. The following sections will discuss the adsorption costs which extends beyond the materials involved in adsorbent preparation.

### Cost per sample volume

Other researchers have recognised that estimating the economic feasibility of the adsorptive removal of contaminants from water using only the cost involved in synthesising the adsorbent is not enough. Few studies have evaluated the cost of magnetic adsorbent to remove contaminants per volume of treated water (Li et al. 2021, 2022a, b; Nozari et al. 2022). Li et al. (2021) estimated the cost of treating wastewater containing 25 mg L^−1^ Sb(III) with amino-functionalised magnetic (MIL-101(Cr)-NH_2_/MnFe_2_O_4_) at a dosage of 223 mg L^−1^, which was found to be 26.24 USD m^−3^. The treatment cost was calculated by multiplying the sorbent dosage by the total production cost of MIL-101(Cr)-NH_2_/MnFe_2_O_4_, as represented in Eq. 1. In a different study, Li et al. (2022a, b) used the same cost estimation method and reported that the treatment of wastewater containing 40 mg L^−1^ of Cd(II) using Fe_3_O_4_/ZIF-8 (MFZ) at a dosage of 141.39 mg L^−1^ was 8.35 USD m^−3^. It is more expensive to treat 1 m^3^ of wastewater using MIL-101(Cr)-NH_2_/MnFe_2_O_4_ than MFZ. The high costs of preparing MIL-101(Cr)-NH_2_/MnFe_2_O_4_, compared to the latter, which is almost two times higher, contribute to this huge difference. In 2022, Li et al., included the costs of raw materials, rinsing solvents, electricity, tap water, and ultrapure water. On the other hand, in 2021, Li et al. used only the cost of chemicals involved in preparing MIL-101(Cr)-NH_2_/MnFe_2_O_4_, which underestimated the total adsorbent cost and the cost to treat 1 m^3^ of wastewater. Noting that the first author of these studies is the same person, and they were published in a year apart, but the parameters used to estimate production of the adsorbents are so different highlights that this part of the research is still developing.

1 $$Treatment\;cost\;(USD)\;per\;m^3\:=\:(adsorbent\;dosage\:\times\:adsorbent\;cost)$$

Recently, Deb et al. (2024) reported that magnetic-halloysite incorporated polymer composite beads (SPHM) removed 10 mg L^−1^ streptomycin for 380 USD per m^3^ of wastewater. This cost is significantly higher than the other costs presented in Table 2. This is despite having the least adsorbent preparation cost out of the four studies that reported adsorbent cost. However, this study employed a different technique to calculate the treatment cost, as shown in Eq. 2. The two equations are very different, which explains the vast cost difference. When the parameters in Deb et al. are substituted in Eq. 1, the cost was 4.07 USD per m^3^ of wastewater, which is lower than that estimated by Li et al. 2021 and Li et al. 2022a.

**Table 2:** The adsorption cost of magnetic adsorbents estimated per volume of contaminated water

Material	Magnetic components	Analytes	Removal/%	Adsorption capacity/mg g^−1^	Cost of adsorbent/USD kg^−1^	Adsorption cost based on sample volume (USD m^−3^)	Ref
Fe_3_O_4_@PAC^a^	Fe_3_O_4_	Dibutyl phthalate	80.1	50.92	-	17.745	(Nozari et al. 2022)
ZA1^b^	Fe_3_O_4_	Gallic acid	-	13.46	-	2.78	(Pirozzi et al. 2023)
MIL-101(Cr)-NH_2_/MnFe_2_O_4_	MnFe_2_O_4_	Sb(II)	-	90.90	117.9	26.24^c^	(Li et al. 2021)
MFZ	Fe_3_O_4_	Cd(II)	-	160.3	59.09	8.35^d^	(Li et al. 2022a)
Cu_x_S/PAD@Fe_3_O_4_^e^	Fe_3_O_4_	Hg(II)	99.9	1395	115	7.70$\times$10^−5^	(Li et al. b)
KFC@Fe_3_O_4_^f^	Fe_3_O_4_	Cs(I)	> 90.0	16.14	-	0.07500	(Su et al. 2023)
SPHM	Fe_3_O_4_	Streptomycin	-	236	8.14	380	(Deb et al. 2024)
Fe_3_O_4_@SiO_2_/ALP-p	Fe_3_O_4_	Bisphenol-A	-	485.1	11.12	0.435^g^	(Zhuang et al. 2024)

^a^Magnetic nanocomposite of Fe_3_O_4_@powdered activated carbon

^b^Magnetic metal-ceramic nanocomposite from zeolite A

^c^Treating 25 mg L^−1^ containing wastewater

^d^Treating 40 mg L^−1^ containing wastewater

^e^Cu_x_S-Fe_3_O_4_-polydopamine nanocomposite

^f^Magnetic potassium ferrocyanide nanocomposite

^g^Treating wastewater containg 50 mg L^−1^ bisphenol-A

2 $$Treatment\;cost\;(USD)\;per\;m^3=(analyte\;dosage/adsorption\;capacity)\:\times\:adsorbent\;cost$$

Zhuang et al. engineered magnetic azo-linked porous polymers (Fe_3_O_4_@SiO_2_/ALP-p) nanocomposite to remove bisphenol-A from wastewater (Zhuang et al. 2024). They discovered that treating wastewater containing 50 mg L^−1^ bisphenol-A costs 0.435 USD m^−3^. This cost was significantly lower than the operating cost when using commercial adsorbents, such as activated carbon (14.1 USD m^−3^) or the developing materials such as graphene and metal-organic frameworks (MOFs), which cost 217.2 USD m^−3^ and 24.3 USD m^−3^, respectively. Interestingly, ALP-p exhibited a cost of 1.28 USD m^−3^ than its corresponding magnetic nanocomposite, Fe_3_O_4_@SiO_2_/ALP-p, which is more than twice as high (Zhuang et al. 2024). This suggests that the magnetic component ensured easy separation, enhanced sorptive active sites, and reduced adsorption cost.

This method of evaluating the treatment cost of magnetic nanoadsorbents, based on the volume of treated water, makes it easy for industries and municipalities to estimate specific costs as treatment vessels have known volume capacities. The volume of the treatment plant can be substituted in the adsorption cost presented in Table 2 to determine the amount of money required to treat the targeted analyte in that volume. The disadvantage of this approach is that different water sources worldwide contain distinct concentration levels of contaminants, and the adsorption costs were calculated using a specific concentration. Therefore, using the values in Table 2 may result in cost overestimations of water sources that contain far lower concentration levels and underestimations in instances where water is more concentrated than the studied water. The former scenario would result in wasteful expenditure, whereas the latter would result in insufficient removal of the target analyte.

### Cost per mass of adsorbate

There are disadvantages to using only the cost of preparing the adsorbents and excluding the removal performance. Only the method reported in Eq. 2, which includes the adsorption capacity, determines the cost of treating 1 m^3^ of wastewater. In Eq. 2, the higher the adsorption capacity of the adsorbent, the lower the cost of treating wastewater per m^3^. Some materials may exhibit higher preparation costs than others, but their superior adsorptive removal performance renders them more cost-effective alternatives in industrial applications. This was highlighted in the study by Nordin et al. (2021), which revealed that the cost of preparing MCPEI (523 USD kg^−1^) was higher than the commercial activated carbon (450 USD kg^−1^). However, they revealed that it would require a large mass of activated carbon to remove anionic reactive black 5 compared to the MCPEI nanocomposite. This is attributed to the significantly low adsorption capacity (71.42 mg g^−1^) compared to the latter (330 mg g^−1^). Consequently, this significantly reduced the application cost of the MCPEI nanocomposite over activated carbon. This analysis emphasised the importance of including adsorption performance when selecting suitable adsorbents in terms of cost-effectiveness.

Table 3 presents studies that employed the method of estimating removal costs per gram of adsorbed analytes. Here, the adsorption costs were calculated by dividing the cost of preparing the adsorbent by the adsorption capacity, as shown in Eq. (3). Adsorbent materials must be cheap and have a large removal capacity (adsorption capacity) for targeted analytes to achieve low adsorption costs. This means the material must exhibit large sorptive sites and functional groups that attract the contaminants to the surface. Most adsorption costs in Table 3 are lower than those estimated based on the cost of treating 1 m^3^ of water (Table 2). This phenomenon is easily reflected in the study by Li et al. (2022a), which reported a cost of 1.86 USD g^−1^ of adsorbed Cd(II), which was much lower than 8.35 USD per m^3^ of treated wastewater. The treatment cost per volume was calculated using 3.3 times less adsorbent preparation cost after factoring in solvent recovery during the preparation stage. When considering solvent recovery, the cost would drastically reduce from 1.86 to 0.582 USD g^−1^.

**Table 3:** The adsorption cost of magnetic adsorbents calculated per gram of treated analyte in water

Material	Magnetic component	Analytes	Removal/%	Adsorption capacity/mg g^−1^	Cost of adsorbent/USD kg^−1^	Adsorption cost (USD g^−1^)	Ref
Fe_3_O_4_@YM^a^	Fe_3_O_4_	Cr(VI)	-	161.3	12.10	0.07500^b^	(Mercado et al. 2023)
Fe_3_O_4_@CD				312.5	43.76	0.1400^b^	
Z@Fe_3_O_4_-MnO_2_^c^	Fe_3_O_4_	Arsenic	> 90	80.0	36.07	0.45	(Angaru et al. 2023)
CaFe_2_O_4_/ZrO_2_-MNC	CaFe_2_O_4_	Metanil Yellow	90.3	454.6	31.74	0.06980	(Bhowmik et al. 2022)
					6.910^b^	0.01520^d^	
		Cr(VI)	> 90	178.6	31.74	0.1780	
					6.910^b^	0.03870^b^	
AMB^e^	Fe_3_O_4_	F^−^	98.1	35.53	11.10	0.310	(Kumar et al. 2023)
MGOCSAC	Fe_2_O_3_ and Fe_3_O_4_	Methylene blue	80.2	270.3	522.9	1.935	(Chan et al. 2024)
MCSAC			76.4	169.5	484.6	2.859	

^a^YM-coated magnetite

^b^Recalculated so that the ratio is USD/g

^c^Binary oxides decorated zeolite

^d^Industrial scale

^e^Activated magnetic biochar

3 $$Adsorption\;cost\;(USD\;g^{-1})\:=\:adsorbent\;cost/adsorption\;capacity$$

Mercado et al. prepared magnetite carbon dots composite (Fe_3_O_4_@CD) for 43.76 USD kg^−1^ to treat Cr(VI) from water (Mercado et al. 2023). The cost is higher than that of preparing CaFe_2_O_4_/ZrO_2_ magnetic nanocomposite (CaFe_2_O_4_/ZrO_2_-MNC) (31.74 USD kg^−1^) to remove Cr(VI) from water, as shown in Table 1 (Bhowmik et al. 2022). However, the adsorption cost for Fe_3_O_4_@CD is lower than that of CaFe_2_O_4_/ZrO_2_-MNC due to its larger adsorption capacity, as shown in Table 3. A similar occurrence was observed in the study by Chan et al., where the preparation cost for graphene oxide/iron oxide/coconut shell activated carbon composite (MGOCSAC) was higher than that for magnetic coconut shell activated carbon (MCSAC) to remove methyl blue from water. However, the adsorption cost was the opposite due to a significantly larger adsorption capacity attributed to the latter, as shown in Table 3 (Chan et al. 2024). This phenomenon suggests that the good adsorptive performance of a magnetic adsorbent can reduce the operational cost and should be included in cost analysis.

Including the adsorptive performance of the material toward targeted contaminants allows for a cost analysis of removing those specific contaminants instead of the general cost of the material. For instance, Bhowmik et al. (2022) used CaFe_2_O_4_/ZrO_2_-MNC to remove Metanil Yellow and Cr(IV) from water; the cost to remove the former is 2.6 times lower than the cost of removing Cr(IV), corresponding to its larger adsorption capacity (2.6 times larger), as presented in Table 3. If the adsorption capacities were excluded, the cost to remove both analytes would have been overestimated to 31,7 USD kg^−1^, and the helpful information regarding CaFe_2_O_4_/ZrO_2_-MNC being favourable and cheaper to treat Metanil Yellow would not have been extracted.

Bhowmik et al. (2022) introduced industrial-scale preparation cost in estimating economic viability, which is an interesting aspect of estimating adsorption cost. They revealed that industrial-scale preparation costs are much cheaper than lab scale costs; in this case, the adsorption cost of Cr(IV) and Metanil Yellow was consequently cheaper, as shown in Table 3. Since the lab scale is a preliminary phase for industrial-scale application, concluding an economic cost analysis at the initial phase might seriously result in underestimation. Including the industrial-scale approach in cost analysis is encouraged, as it will give a realistic economic analysis for industries in terms of selecting the best-performing adsorbent at a cheaper cost.

### Cost recalculated to include adsorption performance

This study used Eq. 3 to convert the costs presented in Tables 1 and 2 into adsorption costs (the removal costs in USD per gram of analyte). This was employed to gain a proper, fair, and uniform analysis of the costs associated with magnetic adsorbent in water remediation studies based on the mass of target analyte being removed; the results are presented in Table 4. This will enable an unbiased comparison of the adsorption costs of materials targeting similar analytes. The results showed that removing a gram of targeted contaminants was significantly cheaper than the adsorbent preparation costs. The studies by Isaac et al. (2023) and Sahare et al. (2023) removed Cu(II) from water. It costs approximately 28 times more to prepare NH_2_-CF-CB than MJBR; however, the adsorption cost for the former was 3 times higher. This phenomenon indicated a significant reduction in costs after including their adsorption performances, as shown in Table 4. A similar phenomenon was observed in the removal of Pb(II) using MagA and MGO@TNs, as presented in Table 4 (Akar et al. 2022; Yang et al. 2020). The preparation cost of MGO@TNs was 4.4 times higher than that of MagA, but its adsorption cost was 2.2 times higher. The reduced costs are due to its excellent adsorption performance compared to MagA. However, the cheapest material to remove Pb(II) was MBC-IO, with a cost of 0.00379 USD g^−1^ and 11 times cheaper than that for MgA, as seen in Table 4 (Latifian et al. 2024). This cost is mainly attributed to its excellent adsorption capacity of 504 mg g^−1^.

**Table 4:** Recalculation of costs in Tables 1 and 2 to include adsorption capacity of the magnetic adsorbent for target contaminants in water

Material	Magnetic component	Analytes	% removal	Adsorption capacity/mg g^−1^	Costs of sorbent/USD kg^−1^	Adsorption cost (USD g^−1^)	Ref
MagA	Fe_3_O_4_	Pb(II)	> 90.0	158.7	6.70	0.0422	(Akar et al. 2022)
MgF-NPs	MgFe_2_O_4_	Malachite Green	> 90.0	487.6	50.88	0.104	(Das et al. 2021)
CT@Fe_3_O_4_	Fe_3_O_4_	U(VI)	99.0	45.50	14.03	0.308	(Das et al. 2023a)
MZ@β-CD-GA	Fe_2_O_3_	Levofloxacin	89–98	145.0	2131.5	14.7	(Mpelane et al. 2023)
MCPEI^f^	Fe_3_O_4_	Anionic reactive black 5	100	330.0	532	1.61	(Nordin et al. 2021)
MNCDES	Fe_3_O_4_	Malachite Green	92.7	87.72	0.00180	2.05 × 10^−5^	(Sadiq et al. 2021)
MnFe_3_O_4_/CS	Fe_3_O_4_	Cu(II)	-	62.30	3.30	0.0530	(Zhang et al. 2021)
		Cd(II)		60.60		0.0545	
MF-CMS	Fe_2_O_3_	Tetracycline antibiotics	85.9–96.5	64.93–94.63	5.91	0.0625–0.0910	(Zhang et al. 2023a)
MIP-MBC	Fe_3_O_4_	Sulfamethoxazole	95.7	25.65	0.100	0.00390	(Li et al. 2023)
MJRB	Fe_2_O_3_/Fe_3_O_4_	Ni(II)	98.1	18.11	1.66	0.0917	(Isaac et al. 2023)
		Cu(II)	98.3	16.83		0.0986	
Zr@MPN-PEI	Fe_3_O_4_	PO_4_^2−^	> 70.0	36.60	0.501	0.0137	(Aryee et al. 2023)
MAG–OSBC	Fe_3_O_4_	Clofazimine	98.6	174.0	126.45	0.727	(El‐Azazy et al. 2021)
SACFe	Fe_2_O_3_	Sulfamethoxazole	95.4	58.44	2850.3	48.8	(Prasannamedha et al. 2022)
BTSFe	Fe_2_O_3_	Ofloxacin	96.0	19.74	2.70	0.137	(Singh and Srivastava 2020)
Fe_3_O_4_@DAL	Fe_3_O_4_	Pefloxacin, lomefloxacin, enrofloxacin, and difloxacin	> 80.0	19.2–33.7	275.58	8.18–14.4	(Hou et al. 2022)
NH_2_-CF-CB	CoFe_2_O_4_	Malachite Green	> 80.0	357.2	47.60	0.133	(Sahare et al. 2023)
		Cu(II)		158.7		0.300	
CoFe_2_O_4_	CoFe_2_O_4_	Thiazole Yellow G	99.0	67.06	267.42	3.99	(Patil and Behera 2023)
		Alizarin Yellow R	93.0	60.19		4.44	
NiFe_2_O_4_	NiFe_2_O_4_	Thiazole Yellow G	96.9	63.04	69.99	1.11	
		Alizarin yellow R	92.0	55.71		1.26	
MgFe_2_O_4_-PANI-NC	MgFe_2_O_4_	Methyl Red	90.2	98.04	100.60	1.03	(Das and Debnath 2023)
FAM-MCA/Fe_3_O_4_	Fe_3_O_4_	Victoria Blue	> 90.0	279	6.64	0.0238	(Sheerazi et al. 2024)
MGO@TNs	Fe_3_O_4_	Pb(II)	99.8	322.7	29.76	0.0922	(Yang et al. 2020)
ZnO-Fe_3_O_4_	Fe_3_O_4_	Methyl blue	-	330.0	1.50	0.00455	(Cheng et al. 2019)
MGO-NH-SH	Fe_3_O_4_	Hg(II)	> 90.0	208.8	38,554	185	(Kazemi et al. 2019)
Fe_3_O_4_@SiO-NH-SH	Fe_3_O_4_		> 90.0	176.8	20,202	114	
MMPC/Cyc-Chit	Fe_3_O_4_	Three fluoroquinolones	> 90.0	130–195	1324.9	6.79–10.2	(Mashile et al. 2020)
MNSB	Fe_2_O_3_	Tetracycline hydrochloride	96.3	143.9	52.59	0.365	(Zhang et al. 2024a)
GSMB2	Fe_2_O_3_	Cr(VI)	-	7.61	2.44	0.321	(Zhang et al. 2024b)
Fe_3_O_4_-RAC	Fe_3_O_4_	Methylene blue	95.2	32.25	22.29	0.691	(Vaddi et al. 2024)
MBC-IO	Fe_3_O_4_	Pb(II)	93.5	504	1.912	0.00379	(Latifian et al. 2024)
POM-MBC	Fe_3_O_4_	Metronidazole	94.3	78.45	80.03	1.02	(Mohammadian et al. 2024)
CHB-Fe-CS	Fe_2_O_3_	Zn(II)	> 85.0	270	10 430	38.6	(Sopanrao and Sreedhar 2024)
γ-Fe_2_O_3_-LSB	Fe_2_O_3_	Methylene blue	98.0	54.55	1.88	0.0327	(Mishra et al. 2024)
PVA-MH	Fe_2_O_3_/Fe_3_O_4_	Cd(II)	95.7	13.8	8.38	0.607	(Karlıdağ et al. 2024)
GSH/Fe_3_O_4_@GO	Fe_3_O_4_	Ag(I)	-	755	1900	2.51	(Zounia et al. 2024)
FCZ@C-600	Fe_3_O_4_	Dimethylhydrazine	-	185.7	660.2	3.56	(Su et al. 2024)
Mag@GAS	Fe_3_O_4_	Tylosin tartrate	80.6	188.6	4.30	0.0228	(Li et al. 2024)
Fe_3_O_4_	Fe_3_O_4_	Cr(VI)	100	91.15	110	1.21	(Yadav et al. 2024)
Fe_3_O_4_/NiO	Fe_3_O_4_		100	150	149.07	0.994	
Pan-MgF-NC	MgFe_2_O_4_	Cationic brilliant green	90.28	294.1	85.36	0.290	(Das et al. b)
Fe-MC3-1000	Fe_2_O_3_/Fe_3_O_4_	Congo red	97.2	972.1	20.83	0.0214	(Zhang et al. b)
AF-Fe_3_O_4_	Fe_3_O_4_	Cr(VI)	-	212.1	16.19	0.0763	(Luo et al. 2023)
DETA/SiO_2_/Fe_3_O_4_	Fe_3_O_4_	Cu(II)	99.0	13.46	1.30	0.0966	(Chen and Chang 2022)
P3ABA/GO/CoFe_2_O_4_	CoFe_2_O_4_	Congo red	98.0	153.9	2906	18.9	(Babakir et al. 2022)
Wheat bran/Fe_3_O_4_	Fe_3_O_4_	Methylene blue and methyl violet	97.5 and 98.8	46.08 and 51.28	8.00	0.174 and 0.156	(Pooladi et al. 2021)
0.3Ma-MgMnLDO-a	Fe_3_O_4_	Cd(II)	99.0	422.6	39.0	0.0923	(Chen et al. 2020)
MIL-101(Cr)–NH_2_/MnFe_2_O_4_	MnFe_2_O_4_	Sb(II)	-	90.90	117.9	1.30	(Li et al. 2021)
MFZ	Fe_3_O_4_	Cd(II)	-	160.3	59.09	0.369	(Li et al. 2022a)
Cu_x_S/PAD@Fe_3_O_4_	Fe_3_O_4_	Hg(II)	99.9	1395	115	0.0824	(Li et al. b)
SPHM	Fe_3_O_4_	Cs(I)	-	236	8.14	0.0345	(Deb et al. 2024)
Fe_3_O_4_@SiO_2_/ALP-p	Fe_3_O_4_	Bisphenol-A	-	485.1	11.12	0.0231	(Zhuang et al. 2024)

The preparation cost for 0.3Ma-MgMnLDO-a was 4.6 times higher than that for PVA-MH (Chen et al. 2020; Karlıdağ et al. 2024). Both of these materials were applied in removing Cd(II) from water, in which 0.3Ma-MgMnLDO-a displayed a greater adsorption capacity than the latter, as shown in Table 4. After calculating the adsorption cost, applying PVA-MH to remove Cd(II) was highly expensive and cost 0.607 USD g^−1^, which is 6.6 times higher than applying 0.3Ma-MgMnLDO-a (Chen et al. 2020; Karlıdağ et al. 2024). This phenomenon further emphasises the importance of incorporating adsorptive performance when determining costs.

Yadav et al. (2024) employed Fe_3_O_4_ and its nanocomposite form, Fe_3_O_4_/NiO, to remove Cr(VI) from water. Fe_3_O_4_ nanoparticles were less expensive to prepare but exhibited a smaller adsorption capacity than the nanocomposite, as seen in Table 4. However, the adsorption cost attributed to the nanocomposite was lower than that for Fe_3_O_4_ owing to a larger adsorption capacity. The increased adsorption capacity associated with the nanocomposite may be attributed to the enhanced adsorption sites due to adding NiO. This assertion is supported by the fact that Fe_3_O_4_/NiO has a larger specific surface area (163.9 m^2^ g^−1^) than Fe_3_O_4_ (110.7 m^2^ g^−1^), which is an indication of increased adsorptive sites for Cr(VI) (Yadav et al. 2024). In this scenario, in as much as the hybridisation of MNPs enhanced the preparation cost, that was redressed by its removal performance, which subsequently reduced the adsorption cost. Moreover, Fe_3_O_4_/NiO showed better regeneration and reusability compared to Fe_3_O_4_, in which after six cycles, the removal percentage was above 70% for the nanocomposite and below 60% for Fe_3_O_4_ (Yadav et al. 2024). The regeneration and reusability further reduced the cost of Fe_3_O_4_/NiO. Therefore, some hybridisation may seem costly at the adsorbent preparation stage, but it may be necessary to curtail the overall adsorption cost when considering the adsorptive performances of nanocomposites.

The heterogeneity in estimation methods discussed in the previous sections, especially those concerning the adsorbent preparation costs, is not resolved by the recalculation done here. The presented adsorption costs in Table 4 still have the inconsistencies discussed; a fair comparison would require comparing studies that include similar parameters in their cost estimation. This can be addressed by developing a standard procedure for estimating adsorbent preparation costs and adsorption costs.

## Factors that must be considered when estimating adsorption costs

### Regeneration and reusability

**Fig. 3:**
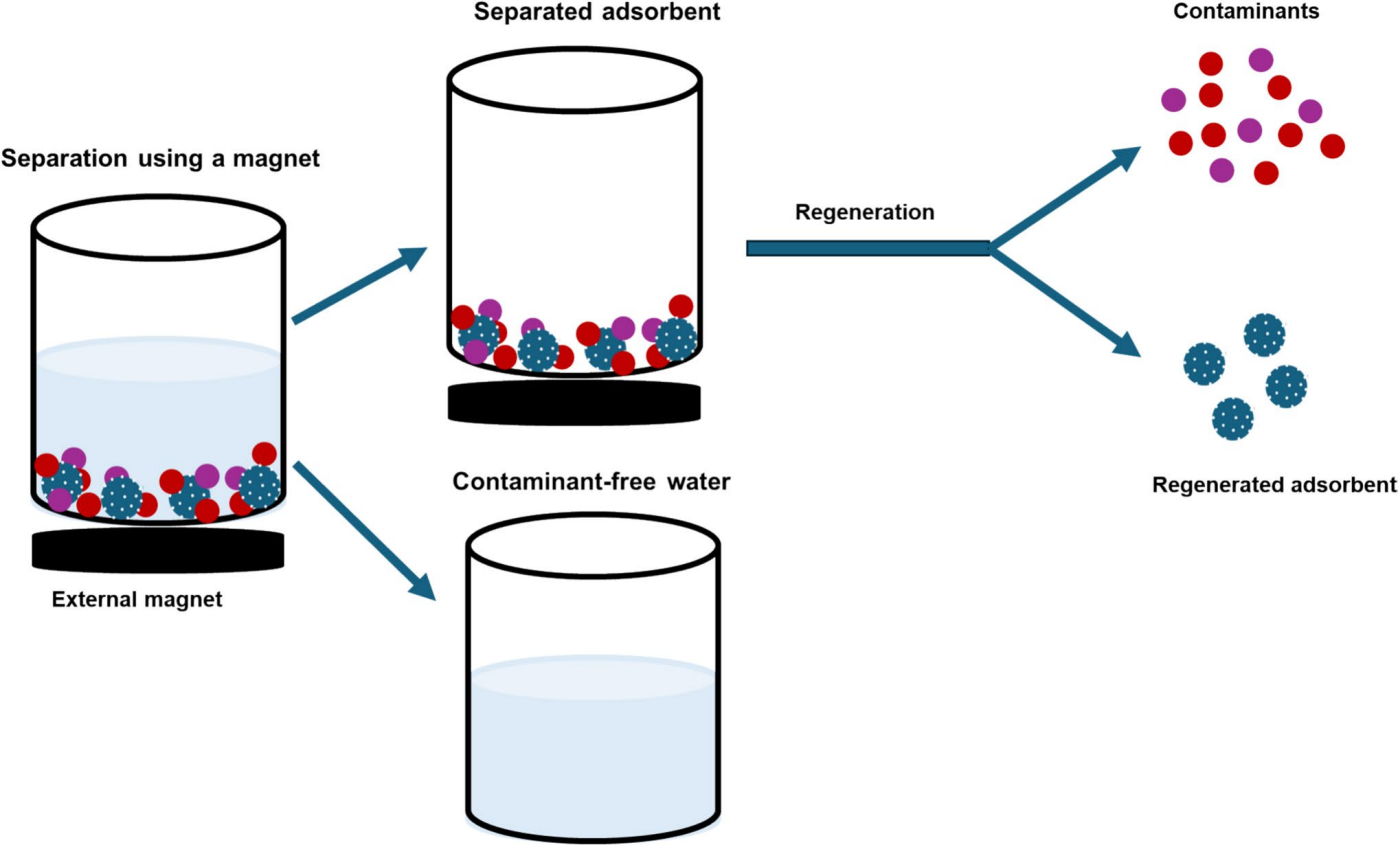
Schematic diagram showing the regeneration of the magnetic adsorbent after adsorption

It is paramount to recover the adsorbent after contaminant adsorption to avoid the disposal of adsorbent-containing pollutants, as this can create secondary pollution. Adsorbent regeneration entails the separation of the magnetic adsorbent from the solution (contaminant-free water) by an external magnet after adsorption, as shown in Fig. 3. The adsorbed contaminants are removed from the magnetic adsorbent through chemical and thermal treatment to regenerate the magnetic adsorbent. Moreover, the regeneration and reusability of the material play a significant part in reducing the adsorption costs. This refers to the ability of a material to be used several times while maintaining high adsorption performance. If a material is reused twice, the overall adsorption costs are reduced two-fold, provided zero cost is incurred during the desorption process. This assertion was supported by Deb et al. (2024), where SPHM cost USD 0.38 to remove 1 m^3^ of wastewater containing streptomycin, which was reduced to USD 0.038 after factoring in the ten-fold reusability of the material. However, this study did not include the cost of 100% ethanol as an eluting solvent for material regeneration; thus, the cost may be slightly underestimated. In a different study, Nozari et al. (2022) reported a reduction to half of the cost of Fe_3_O_4_@PAC due to high removal performance and reusability in the five adsorption-desorption cycles. The regeneration and reuse of adsorbents also reduce the generation of waste associated with the disposal of the material after adsorption, which can negatively impact the environment via secondary pollution. However, some waste may still be generated as eluting solvents; this aspect in reusability studies, including the desorption cost, is worth investigating.

The desorption of analytes from the material after adsorption also incurs costs due to the solvent system commonly used. Deb et al. (2024) and Nozari et al. (2022) employed ethanol and sodium hydroxide, respectively, to elute analytes and free the magnetic adsorbents. Li et al. (2022a, b) reported that solvents could be recovered at a rate as high as 90% in industrial output, and the recovery of the cleaning solvent was factored into the cost estimation of the treatment cost. This factor can be explored by recovering and reusing eluting solvents and recovering the analytes to be sold as secondary products. This phenomenon was demonstrated by Oladipo and Gazi (2016), who sold boric acid after the extraction of boron using a lignocellulosic magnetic hybrid as an adsorbent. Singh and Srivastava (2020) utilised thermal desorption to remove ofloxacin and recovered BTSFe with temperatures up to 350 ℃. Although this method circumvents the cost of eluting solvent and waste generation, it may incur expenses similar to or more than elution costs due to the energy of the oven and muffle furnace. Ultrasonic regeneration is a cheaper alternative; it is safe and simple and consumes less energy (Gkika et al. 2025).

There are limited studies that investigated the regeneration and reusability of adsorbents, further to calculate the cost implications in the treatment process. In the near future, the economic analysis presented in Table 4 should include the regeneration and reusability of the magnetic adsorbents to better compare the cheapest and most reusable materials.

### Contact time

Contact time is a very important factor when designing a water remediation process. The duration to completely remove contaminants from water should be reasonably short for the adsorbent to be applicable in industrial and municipal water treatment systems. No matter how cheap the adsorption cost may be, if the materials take several hours (e.g. 24 h) to adsorb the contaminants, that system is impractical to implement in real-life situations and will tend to be costly when the energy cost associated with the adsorption duration is included. For example, Youssif et al. (2024) fabricated and employed Fe_2_O_3_@SiO_2_ nanocomposite to remove Hg(II) from water and the material reached equilibrium in 180 min. Conversely, Hg(II) adsorption with MGO-NH-SH and Fe_3_O_4_@SiO-NH-SH reached equilibrium within 60 min, three times shorter than 180 min obtained with Fe_2_O_3_@SiO_2_ (Kazemi et al. 2019). Both studies used a shaker to agitate the mixture of materials and the Hg(II) solution, thereby facilitating adsorption. This means it would cost three times more energy to remove Hg(II) with Fe_2_O_3_@SiO_2_ and contributes to increasing the overall energy consumption cost.

Contact time is highly dependent on the functional groups and textural properties of the material, which directly influence the adsorptive mechanism between the adsorbent and the targeted pollutant. For instance, mesoporous materials ensure fast diffusion of contaminants from water during adsorption, thus shortening the removal time. Additionally, parameters such as sample pH need to be investigated to determine optimum conditions to reduce contact time. This is because the sample pH affects the interaction between the analytes and magnetic adsorbents. Moreover, optimum pH may enhance adsorption rates, reduce the mass of adsorbent, and subsequently, overall adsorption cost may be reduced. The effect of contact time does not need to be calculated separately and incorporated into the total cost; it should form part of the energy cost contribution to the material preparation and adsorption process. This process might include instruments like an ultrasonicator, a shaker, and a vortex as a technique that facilitates adsorption. As noted in the previous sections, energy costs significantly affect the overall cost of the material/adsorption process; as a result, investigating the contact is of paramount importance in designing cost-effective water remediation systems.

## Worked example of a typical good cost estimation method

Here, the study by Bhowmik et al. (2022) was employed to demonstrate a step-by-step calculation of the adsorption cost of Metanil Yellow dye (MY) and Cr(VI) from water using CaFe_2_O_4_/ZrO_2_-MNC. Additional factors and calculations were added from the original study to show a typical example of a good cost estimation technique. Table 5 presents a list of chemicals and instrumentation used to prepare CaFe_2_O_4_/ZrO_2_-MNC for determining the synthesis cost. The net costs and total adsorbent costs were calculated using Eqs. (4) and (5), respectively.

**Table 5:** Breakdown of costs of reagents and energy used in preparing CaFe_2_O_4_/ZrO_2_-MNC (Bhowmik et al. 2022)

Parameters	Unit cost (lab scale) (USD)	Unit cost (industrial scale) (USD)	Quantity	Net cost (lab scale) (USD)	Net cost (industrial scale) (USD)
FeCl_3_•6H_2_O	4.02/500 g	0.25/500 g	32.92 g	0.26	0.017
CaCl_2_•2H_2_O	15.96/500 g	0.091/500 g	14.70 g	0.47	0.0027
ZrOCl_2_•8H_2_O	6.14/500 g	1.03/500 g	32.22 g	0.40	0.066
NaOH	4.34/500 g	0.21/500 g	35.0 g	0.30	0.015
Oven drying	0.079/kWh	0.079/kWh	9 kWh (1.5 kW)	0.0065	0.0065
Calcination (muffle furnace)	0.079/kWh	0.079/kWh	12 kWh (2 kW)	0.40	0.40
Net preparation cost (80 g)				22.96/kg	6.40/kg

4 $$Net\;cost\;(USD)\:=\:Unit\;cost\:\times\:quantity$$

5 $$Net\;adsorbent\;preparation\;cost\;(USD/kg)\:=\:(net\;reagent\;costs)\:+\:(net\;energy\;costs)/mass\;of\;adsorbent\;(80g)$$

Table 6 represents the adsorption capacities and energy used to facilitate the adsorption of MY and Cr(VI) to estimate their adsorption costs. The net adsorption cost of MY and Cr(VI) utilising CaFe_2_O_4_/ZrO_2_-MNC was calculated using Eq. (6).

**Table 6:** Adsorption cost of MY and Cr(VI) by CaFe_2_O_4_/ZrO_2_-MNC (Bhowmik et al. 2022)

Pollutant	Adsorption capacity (mg/g)	Energy cost (adsorption) (USD)	Net adsorption cost (lab scale) (USD/g)	Net adsorption cost (lab scale) (USD/g)
MY	454.55	0.0036^a^	0.054	0.018
Cr(VI)	178.57	0.014^b^	0.14	0.050

^a^Ultrasonic bath

^b^Batch stirrer

6 $$Net\;adsorption\;cost\;(USD/g)\:=\:((Net\;adsorbent\;cost\:+\:energy\;cost\;(adsorption))/adsorption\;capacity$$

The regeneration and reusability study was factored into the adsorption cost of MY and Cr(VI). The adsorption-desorption of contaminants was conducted for five cycles and produced removal performance above 80% for MY and Cr(VI) (Bhowmik et al. 2022). The adsorption cost, including regeneration cycles, was calculated using Eq. (7). However, the study did not include the cost of reagents and instruments employed for the adsorption-desorption cycles. Therefore, these were included in the overhead cost. The overhead cost was employed to account for parameters (such as water costs and reagents used in the regeneration study) that were not included by the authors. The net adsorption cost, inclusive of overhead cost, was estimated using Eq. (8).

7 $$Net\;adsorption\;cost\;(after\;regeneration)(USD/g)(D)\:=\:net\;adsorption\;costs/number\;of\;cycles\;(5\;cycles)$$

8 $$Net\;adsorption\;cost\;(plus\;10\%\;overhead)(USD/g)\:=\:net\;adsorption\;cost(D)\:+\:10\%\;of\;net\;adsorption\;cost(D)$$

**Fig. 4:**
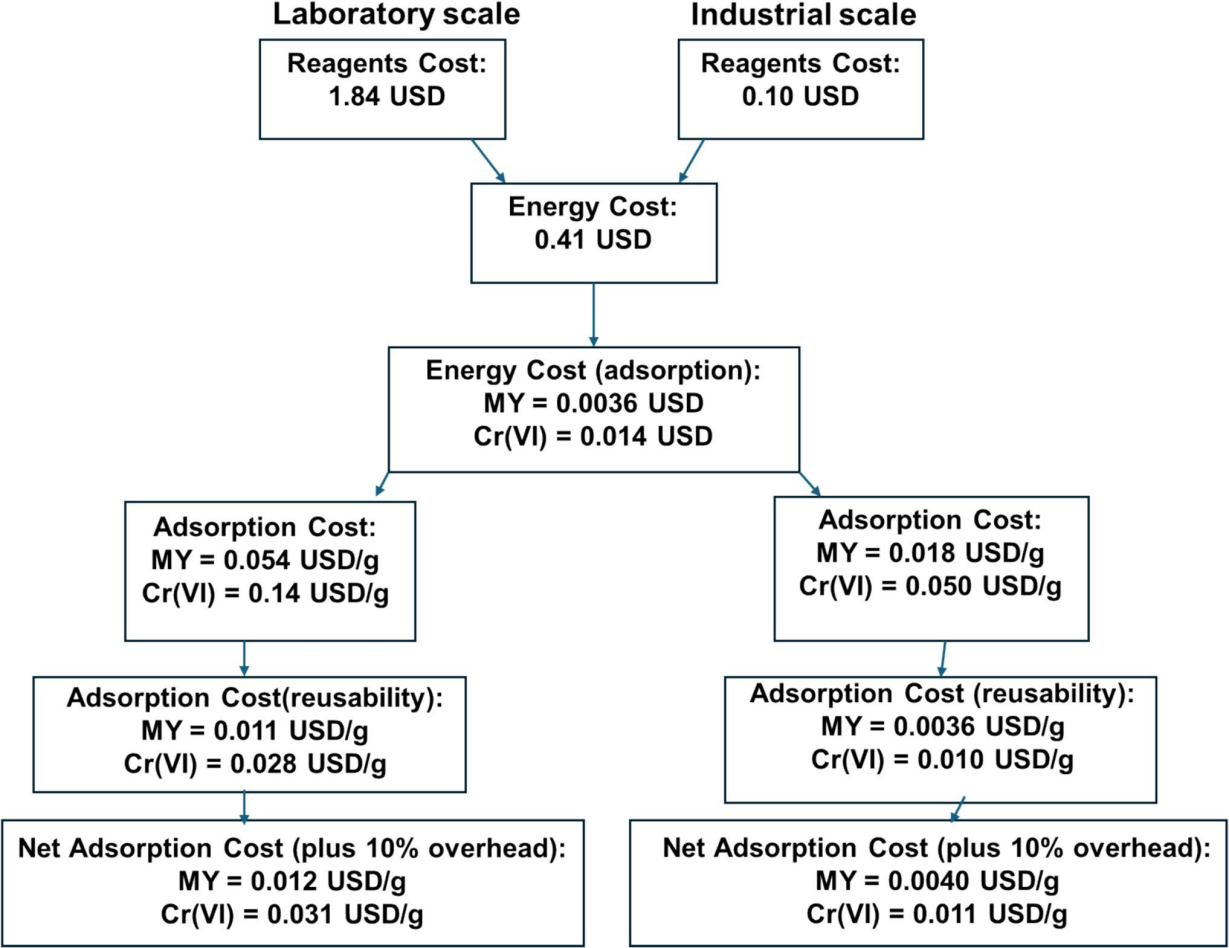
Flow diagram showing step-by-step cost estimation of removing MY and Cr(VI) using CaFe_2_O_4_/ZrO_2_-MNC

Figure 4 shows a schematic representation of the step-by-step calculations of the adsorption cost of MY and Cr(VI) using CaFe_2_O_4_/ZrO_2_-MNC nanocomposites. It was demonstrated that the adsorbent reusability factor significantly reduced adsorption cost (Fig. 4).

**Fig. 5:**
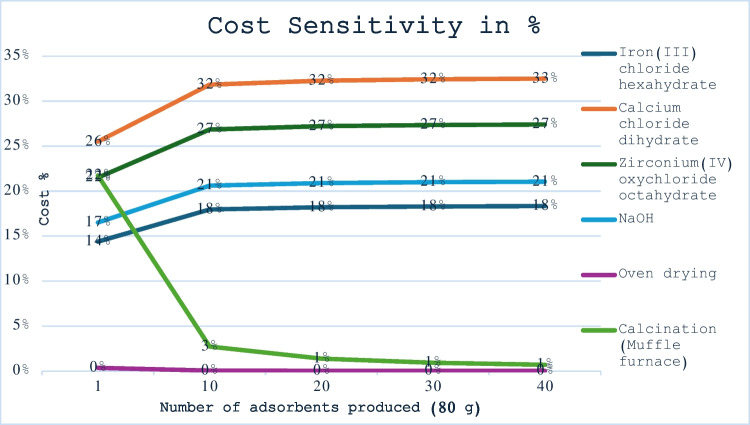
Cost sensitivity assessment of parameters involved in preparing CaFe_2_O_4_/ZrO_2_-MNC

Cost sensitivity assessment for the real case study discussed above was employed to determine parameters that have significant impact on the cost of CaFe_2_O_4_/ZrO_2_-MNC. The assessment was investigated to determine how percentage cost of each parameter changes with increased production of the adsorbent and the results are shown in Fig. 5. The results showed that the parameters with significant influence in the cost of adsorbent increased in the order of oven drying, muffle furnace, FeCl_3_•6H_2_O, NaOH, ZrOCl_2_•8H_2_O, and CaCl_2_•2H_2_O. The oven drying and muffle furnace are cost parameters not positively affected by the increase in adsorbent production; hence, their influence was the lowest.

Table 7 shows the dos and don’ts in ensuring the prepared magnetic adsorbent is cheap and the removal of pollutants from environmental water and wastewater is economical and sustainable.

**Table 7:** Dos and don’ts in adsorption cost analysis

Factors	Dos	Don’ts
Transport	Include transport costs for raw materials	Transport in bulk to cut costs
Water	Include municipal rates	Avoid ultrapure water to cut costs
Reagents	Buy reagents in bulk	Avoid analytical grade reagents to reduce costs
Energy	Use mechanical stirring and grinding	Avoid synthetic methods that require multiple energy-intensive steps
Reusability	Select materials that are stable and regeneratable	Choose regeneration methods that are reagent free
Upscaling	Industrial scale should be included to cut costs	Avoid scaling up that increases energy consumption
Functionalisation/hybridisation	Use easily accessible and abundant waste to form magnetic nanocomposites	Avoid using expensive precursors
Contact time	Ensure functional groups of the material have high affinity to target analytes to enhance adsorption kinetics	Adsorbent-analytes interaction that takes hours to reach equilibrium should be avoided

## Conclusion

Magnetic nanoparticles and magnetic nanocomposites are widely used in water remediation due to their excellent performance and ease of separation. This study highlighted some of the preparation methods, namely co-precipitation (Massart method), thermal decomposition, and microwave-assisted techniques, for MNPs since they also contribute to the overall cost of the adsorption. Thermal decomposition and microwave-assisted methods use more energy-intensive instruments than co-precipitation, but they yield highly crystalline, uniform, and smaller particle sizes. This study also examined three cost analysis approaches: estimation based on chemical costs, adsorption cost per unit sample volume, and adsorption cost per gram of removed contaminant. Approximately 76% of the reviewed studies employed chemical cost estimation because of its simplicity; however, this approach is the least accurate and least suitable of the three. Among the methods discussed, the adsorption cost per gram of removed contaminant is the most robust, as it directly incorporates the adsorption performance of the material through its adsorption capacity. This was illustrated by the substantial reduction in the cost of methyl blue removal using ZnO-Fe₃O₄, which decreased from 1.50 USD kg^−1^ (based on adsorbent cost) to 0.00455 USD g^−1^ (based on adsorbate removal) when a high adsorption capacity of 330 mg g^−1^ was considered. Furthermore, a worked example demonstrated that accounting for adsorption capacity, adsorbent regeneration and reusability, and industrial-scale operation leads to a significant reduction in cost and a more realistic economic assessment. In this example, the cost of removing Metanil Yellow dye decreased from 22.96 USD kg^−1^ (laboratory-scale adsorbent cost) to 0.0040 USD g^−1^ (industrial-scale adsorbate removal cost). Therefore, the inclusion of adsorption capacity, regeneration, and industrial-scale considerations is essential for obtaining an accurate and meaningful cost evaluation.

## Data Availability

This study did not use any data.
